# What Factors Affect Patient Satisfaction in Public Sector Hospitals: Evidence from an Emerging Economy

**DOI:** 10.3390/ijerph16060994

**Published:** 2019-03-19

**Authors:** Abid Hussain, Muhammad Safdar Sial, Sardar Muhammad Usman, Jinsoo Hwang, Yushi Jiang, Awaisra Shafiq

**Affiliations:** 1 School of Public Affairs, Zijingang Campus, Zhejiang University, Hangzhou 310058, China; abidhusssain02@gmail.com; 2 Department of Management Sciences, COMSATS University, Islamabad 44000, Pakistan; safdarsial@comsats.edu.pk (M.S.S.); susman@comsats.edu.pk (S.M.U.); 3 The College of Hospitality and Tourism Management, Sejong University, 98 Gunja-Dong, Gwanjin-Gu, Seoul 143-747, Korea; 4 School of Economics and Management, Southwest Jiaotong University, Chengdu 610031, China; 5 Economics department, Bagdad Campus, The Islamia University, Bahawalpur 63100, Pakistan; awaisra017@gmail.com

**Keywords:** measuring patient satisfaction, pharmacy services, laboratory services, doctor-patient communication, physical facilities

## Abstract

Patient satisfaction can identify specific areas of improvement in public sector hospitals. However, the Pakistani healthcare system, and quality of service delivery is rarely assessed through the perspective of patient satisfaction. Our study demonstrated the performance of public healthcare systems in Pakistan by interacting with physical services (tangible and environmental), doctor–patient communication, and pharmacy and laboratory services based on patient satisfaction. Primary data were collected from the patients by using a random sampling method. Patients who participated in the study were visitors of public hospitals’ outpatient departments. A total of 554 questionnaires were circulated, and 445 were received. The confirmatory factor and multiple regression analyses were employed to analyze the collected data. The results revealed that laboratory, as well pharmacy services, had positive significant effects (*p* = 0.000) on patient satisfaction, while doctor–patient communication (*p* = 0.189) and physical facilities (*p* = 0.85) had an insignificant relationship with patient satisfaction. Therefore, it is suggested that a significant communication gap exists in the doctor–patient setting, and that Pakistan’s healthcare system is deprived of physical facilities. Consequently, such services need further improvements.

## 1. Introduction

Developing nations are making every effort to entitle their citizens to receive fundamental healthcare because of limited consumer resources [1]. Public hospitals play an essential role in providing health facilities or services for citizens who seek fundamental medical service, while the outpatient department (OPD) role is very effective [2]. A world without basic health facilities would be a world of despair and suffering. Hypothetically speaking, if the world had a fundamental healthcare system that was universally recognized as providing low quality, that would only incur greater misery and suffering. In this hypothetical system, the cost of healthcare would further burden and intensify, increasing patient concern and worry. A healthcare unit is useless if it is inadequate to decrease patients’ distress. The healthcare sector of any country is a vital pillar for its overall progress, because it also influences all the other sectors, such as medical, moral, political, social, and business. Moreover, it has a number of economic implications [3].

Healthcare is one of the major sustainable development goals (SDGs) of agenda 2030 of the United Nations Organization (UNO), which states that: “ensure healthy lives and promote well-being for all at all ages”, as well as its target to achieve universal health coverage, affordable medicine, accessibility, effective and safe quality, and access to quality essential healthcare for everyone [4]. Even if you are wealthy, every aspect of life if is nullified without good health. The developed world is steadily building their health sectors by making revenue for their national economy by encouraging health tourism. Contrarily, the policy makers of developing countries, such as Pakistan, are helping the health sector of developed countries by seeking their services frequently at the expense of taxes being paid by their own citizens [5], instead of forming their own healthcare system.

In emerging nations like Pakistan, healthcare problems receive more attention from researchers than from policy makers [5], which is a matter of grave concern in Pakistan, because the current average cost of healthcare for the people of Pakistan is 66.5%, far greater than the world average of 18.147% [6]. It is a regrettable situation that the number of dissatisfied patients receiving healthcare in Pakistan’s public hospitals has continuously increased [7,8]. Moreover, a large number of Pakistani citizens are in despair believing that the country’s healthcare sector is severely damaged by corruption [9].

In accordance with these factual figures, it has become increasingly necessary to revise and assess the performance of feebly performing, less developed countries and to counterbalance their performance with that of their counterparts in the developed world. This is of the utmost importance, being a matter of life or death to the citizens of such countries. Javed and Ilyas [5] stated that “despite this despondent situation of healthcare in Pakistan, where quality initiatives are relatively more visible in the manufacturing, education and agriculture sectors than in the healthcare sector, not many scientific studies have been done in this context.” In light of present situation, the perceived performance of public hospitals in Pakistan has been assessed by measuring the satisfaction of Pakistani patients from the public sector. To achieve this, we took into account their opinion concerning four areas of healthcare services; these include: Physical services (tangible and environmental), doctor–patient communication, pharmacy services, and laboratory services. Our study assessed patient satisfaction of those using OPD services in Pakistani hospitals. There is currently a significant gap in the existing literature regarding the evaluation of patient satisfaction with respect to doctor-patient communication, physical facilities, laboratory services and pharmacy services aspects. The current study is specifically useful for assessment of the Pakistani healthcare system, which is commonly associated with the lack of patient satisfaction and quality of service delivery.

**Figure 1:**
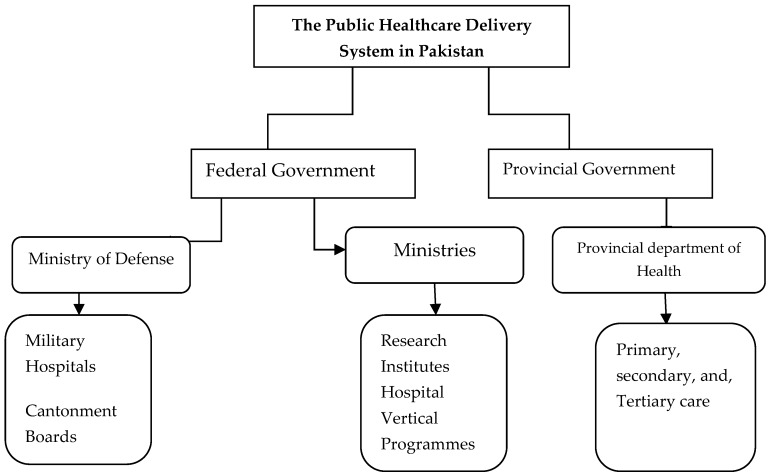
The public healthcare delivery system in Pakistan.

Pakistan has a mixed health system that includes: Public, parastatal, private, civil society, philanthropic contributors, and donor agencies. In Pakistan, healthcare delivery to the public is systematized through four modes of preventive, promotive, curative, and rehabilitative services [10]. According to the constitution of Pakistan, it is the responsibility of the state to provide fundamental health facilities to its citizens without any cost. However, after the implementation of the 18th amendment in the constitution of Pakistan, this duty was shifted from the central government to the provincial governments, who are assumed to devise provincial healthcare policies that affect around 200 million individuals in Pakistan [11]. The public healthcare system of Pakistan is summarized in Figure 1. Central and state governments have their own independent public healthcare setups [12].

## 2. Literature Review

### 2.1. Patient Satisfaction

The notion of patient satisfaction can be evaluated as a multi-faceted approach that mirrors the patient’s experiences when looking for healthcare [13]. When correctly assessed alongside the results and the conformity of treatment, patient satisfaction can be an important tool when assessing the quality and outcome of a particular healthcare setup [14].

Patient satisfaction is a crucial phenomenon that recognizes the patients’ needs so as to improve healthcare systems. A patient that reports satisfaction with their own healthcare is more likely to report better health. Patient responses to healthcare services are one way to obtain information about patient views regarding the perceived quality of healthcare, and to establish robust patient engagement [15]. Patients who report higher satisfaction are more likely to benefit from their treatment [16]. Several scholars have used these factors, which include: Pharmacy services, physical services (tangible, environment), doctor–patient communication, and laboratory services to access the sustainability of healthcare services with a concern for patient satisfaction [17,18,19,20].

### 2.2. Physical Facilities

Physical facilities measure the patient’s perception about the quality of service in regard to the hospital’s physical services. This measure includes: The cleanliness and maintenance of the facility, the availability of physical facility, such as resident rooms, technological capability, diagnostic test rooms, blood banks, wards, beds, ambulance services, waiting rooms, and operation theatres. Several studies have already attempted to ascertain the influence of the physical services in quality delivery [21,22,23].

Forming a pleasant environment that strongly facilitates the patients to make a full recovery. It is very important that the appropriate healthcare staff must work to improve the physical environment of the hospital, such environment will immensely be helpful for the patients to recovery on time and enjoy a healthy life. [20,24].

**Hypotheses** **1** **(H1).** *There is a significant and positive relationship between physical facilities and patient satisfaction.*

### 2.3. Doctor–Patient Relationship

The doctor–patient relationship is important because patients normally ascertain their general perception about healthcare service and its potential outcomes [25,26]. In-order to assess the healthcare services by the patients they must develop a good and friendly relationship with the doctor by using a better level of communication [27]. A satisfactory relationship between doctor and patient is important to ensure that the patient adheres to the medical guidance they have received, which ultimately improves treatment efficacy and reduces transaction expenses [28,29]. This interaction may also boost the patient’s trust and willingness to use such medical facilities at a later date. This repeat usage can also heighten the reputation of the hospital [30,31].

Some studies have also shown that the level of patient health has a direct impact on their level of satisfaction [32,33], with patients in need of more in-depth medical attention, having a stronger impression of the quality of the doctor’s service, alongside their own overall satisfaction. Mekoth, et al. [34] demonstrated that good examination and communication skills of a doctor during outpatient facilities could influence patient views or satisfaction. Yi-ren and Peng-de [35] asserted that five productive underlying aspects included: Hospital staff competency and techniques, their medical effectiveness, the environment they create and the support they offer, the overall attitude and service proceedings. The United Nations organization has recently established sustainability development goals (SDGs) that focus on the importance of a better delivery of service by service providers [4].

**Hypotheses** **2** **(H2).** *There is a significant and positive relationship between doctor–patient communication (relationship), and patient satisfaction.*

### 2.4. Laboratory Services

The key recipient of public healthcare is the patient. As a client of healthcare, the patient is the main focal point of the healthcare delivery system [36]. A patient’s viewpoint regarding the medical care system appears to have been mainly overlooked by the healthcare administrators in less developed countries [37].

Patient satisfaction consists of several elements, for example the quality of clinical facilities provided, pharmacy services, the accessibility of medication, the quality of communication (the attitude of doctors and paramedical employees), institutional infrastructure, physical ease, emotional backing, and respect for client preferences. Disparity among patient anticipation and the healthcare facilities obtained relates to a decline in levels of satisfaction [38]. As a result, assessing patient viewpoints offers them a voice, which can make personal and government owned healthcare services more responsive to the needs of patients [39]. Therefore, efficient laboratory services, that deliver services within a timely fashion, is essential both from a medical and business viewpoint, as well as its impact on patient satisfaction [19].

**Hypotheses** **3** **(H3).** *There is a significant and positive relationship between laboratory services and patient satisfaction.*

### 2.5. Pharmacy Services

The development of new and innovative technological methods and the increased need for specialized healthcare services have brought about a distinct transformation in pharmacy services [40,41,42]. In 2011, the International Pharmaceutical Federation (FIP) and the World Health Organization (WHO) jointly recommended good pharmacy practice guidelines [43]. Consequently, pharmacists have been increasingly involved in marketing their services with a focus on patient satisfaction. Furthermore, pharmacists have been persuaded to become mutually accountable for healthcare consequences and the enhancement of the patient’s quality of life [17,44,45].

However, the clinical training of pharmacists is not advanced, and the activities that represent the usual practice of pharmacists are still executed, in most cases, without direct interaction with the patient [46,47]. Yet, the education of Pakistani pharmacists has endured principal changes in recent years with respect to a professional focus on learning clinical skills. Descriptions of these skills, and the necessary changes that need to be made to the curriculum guidelines of Pakistani institutions are currently being discussed [48].

Pharmacy staff are in a position to clearly define how the patient should use the medication they have been prescribed. Several studies have indicated that these factors are associated with patient satisfaction [48,49,50]. The pharmacy is an important physical service within a hospital, where patients get information about medication [50].

**Hypotheses** **4** **(H4).** *There is a significant and positive relationship between pharmacy services and patient satisfaction.*

## 3. Methodology

### 3.1. Study Setting

Punjab province is the most populous province in Pakistan. It consists of 9 divisions. Bahawalpur division is an administrative division of the Punjab province. It is the largest division of Punjab province and consists of three districts, Bahawalpur, Bahawalnagar, and Rahim yar khan. It consists of 45,588 (km^2^) and its total population is 11,464031. The Punjab has 23 tertiary hospitals and Bahawalpur division has 2 tertiary care hospitals; one is Qaid e Azam Medical College (QAMC), Bahawalpur, that consists of 1600 beds and its affiliated hospital consist of 400 beds; the second hospital is Sheikh Zayed Medical College (SZMC), Rahim Yar khan, that consists of 841 beds, as well as Bahawalnagar has one district hospital [51]. The population of Punjab province and the study area used in this research is exhibited in Table 1. The hospitals used in this study are shown in Table 2.

### 3.2. The Sample

The primary data were collected from the Southern Punjab hospitals by using a random sampling method from June to July 2018. The sample size was 554, as recommended by Saunders [52]. This sample size is justified by the fact that it is in accordance with the size of the population. The questionnaire was divided into two portions. The first portion dealt with demographic characteristics, and the second part measured the quality of services delivery, which included the pharmacy services, the laboratory services, doctor–patient communication, and physical facilities. 445 questionnaires were selected for analysis. The remaining questionnaires were not used for analysis due to unfilled and/or inconsistent answers.

### 3.3. Data Collection and Instruments

The questionnaire for the present research contained 4 factors. The measurement for patient satisfaction consisted of 9 items [53]. The sample item was “The medical care, I have been receiving is just about perfect” and Alpha reliability for this scale was 0.966. Pharmacy services included 8 items [54], and the sample question was “The pharmacist is good at explaining things in a way that I understand.” with Alpha reliability of 0.931. Laboratory services had 6 items [54] with a sample item of “I received the test results in time” and Alpha reliability of 0.927. Physical facilities included 5 items, and doctor–patient communication consisted of 4 items [55]. Sample items for physical facilities and doctor–patient communication were “There was a pleasant atmosphere in the ward” and “The doctors were willing to answer any questions”, respectively. The Alpha reliabilities for these two scales were 0.968 and 0.942, respectively. Patient demographic characteristics included in this study consisted of: Marital status, age, education, occupation, and family income, which are shown in Table 3. The instruments that we used to assess service features from the patients’ perspective were recorded on a five-point Likert’s scale. Following the guidelines from Brislin [56], the questionnaire was translated into Urdu and then translated into English after it was completed. This process was conducted to check for consistency. The questionnaires from illiterate patients were filled out with the assistance of a researcher.

## 4. Results

### 4.1. Sociodemographic Characteristics

The number of males was 44.7%, and the number of females was 55.3%. Overall, 30.8% of respondents were aged 40 to 49, whereas 8.1% were younger than 20 years. In regard to marital status, 54.6% were married and 42.3% were single, while 2.7% were not living with their partner/ husband. Overall, 0.4% was widowed participants. In regard to education and income, about 32.6% had an elementary school level of education, and 27.6% belonged to the category receiving a monthly income of less than 10,000 PKR, while 8.3% received more than 30,000 PKR. In regard to profession, 26.2% were housewives, and 1.6% was students (see Table 3).

### 4.2. Statistical Analysis and Data Interpretation

The data were analyzed using SPSS and AMOS version 24.0. A consistency analysis was performed to assess the reliability of individual variables. Reliability refers to the instrument’s ability to provide consistent results in recurring uses [57]. The Cronbach alpha coefficient has been widely used as a measure for reliability [58]. The results revealed that all variables’ reliabilities (α) exceeded the acceptable threshold value of 0.70 (patient satisfaction = 0.96, laboratory services = 0.92, pharmacy services = 0.93, doctor–patient communication = 0.94, and physical services = 0.96) as suggested by [59,60]. Furthermore, almost all the variables were correlated and statistically significant. See Table 4 for a summary of the descriptive statistics and the zero order correlations and alpha coefficients.

Furthermore, internal consistency was also tested by measuring composite reliability, discriminant validity, and convergent validity for each factor to support more authenticity for the data and the model (see Table 5). Composite reliability (CR) for each factor ranged from 0.781 to 0.923, which exceeded the cutoff criteria (CR > 0.7) [61], while the values of the average variance extracted (hereafter AVE) for each loaded construct ranged from 0.543 to 0.744. This showed convergent validity, because it was shown to be greater than 0.50, which is often used as a threshold [62]. To measure discriminant validity, each value of the square root of the AVE was greater than all the inter factor correlations, as recommended by Shaffer, et al. [63]. All items used in this study had a significant level of (*p* < 0.001).

Based on the exploratory factor analysis (EFA) of the patients’ overall satisfaction, we conducted a confirmatory factor analysis (CFA), and the model fit indices are shown in Table 6. The values of the standardized root mean square residual (SRMR) = 0.042, the normed fit index (NFI) = 0.959, the root mean square error of approximation (RMSEA) = 0.045, the comparative fit index (CFI) = 0.980, the relative fit index (RFI) = 0.951, the incremental fit index (IFI) = 0.980, and the Tucker-Lewis index (TLI) = 0.976. All these values are above, or at, recommended standards [61,64,65,66].

### 4.3. Confirmatory Factor Analysis (CFA)

The confirmatory factor analysis (CFA) confirmed the relationship among the observed variables and their underlying latent variables. It was used to assess the loadings of items on a particular factor. The values of the factor loadings, while conducting the confirmatory factor analysis, are given in Table 7. The statistical values of the confirmatory factor analysis all met the criteria standards for the adequacy of fit. The values of factor loadings showed the strength of relation of the factors with their respective constructs. The maximum value for factor loading is 1. The highest factor loading value in this model was 0.925, and the lowest value was 0.709. All the values of factor loading confirmed the validation of the construct validity, which was measured by inspecting the loadings of the items and their reliability.

### 4.4. Hypothesis Testing Using a Multiple Regression Analysis

Table 8 shows the multiple regression analysis that determined all predictors of overall patient satisfaction in public sector hospitals. According to the results, 37.2% (Adjusted *R*^2^ = 0.367, *F* = 74.33, and *p* = 0.0001) of the variance in the outcome variable (patient satisfaction) was described by the four independent variables, which included pharmacy services, laboratory services, doctor–patient communication, and physical facilities.

To test multi-collinearity, the range of tolerance values was between 0.766 and 0.900 (a value closer to zero indicates a collinearity issue), while the range of the variance inflation factor (VIF) was between 1.238 and1.511 (a value greater than three indicates a problem with collinearity), which showed that multi-collinearity did not exist in the data. The values shown in Table 8 demonstrate that two out of four variables were significant and affected patient satisfaction positively, while the values of two variables were insignificant. Moreover, pharmacy services β = 0.287; *t* = 9.591and *p* < 0.05) showed that one unit increased by 0.232 units in patient satisfaction. Laboratory services (β = 0.331, *t* = 7.713, and *p* < 0.05) revealed that one-unit increase in laboratory services resulted in a 0.256 unit increase in patient satisfaction. Doctor–patient communication (β = 0.055, *t* = 1.136, and *p* > 0.05) and physical facilities (β = 0.076, *t* = 1.727, and *p* > 0.05) were insignificant in this study. The most significant and effecting predictor was laboratory services. Based on these results, H1 and H2 were supported, but H3 and H4 were not supported.

## 5. Discussion and Implications

This research was carried out to examine the service delivery of OPDs in southern Punjab hospitals, Pakistan, from the patients’ perspective. The present study sought to investigate how pharmacy services, laboratory services, doctor–patient communication, and physical facilities measured patient satisfaction alongside the quality of the hospital service. Our results confirmed that patient satisfaction was highly dependent on pharmacy services. such results are similar to those concluded from India, that pharmacy services have a significant impact on patient satisfaction [67]. In a Pakistani context, the pharmacy staff are usually less qualified, a general lack of professionalism, a lack of knowledge regarding the rules and responsibilities of pharmacists and the execution of their duties are the key factors which impacted negatively on the quality of pharmacy services offered by public hospitals [48]. These issues negatively impact on patient satisfaction. The information that pharmacists provide to patients is insufficient, which leads to a deficient interaction between the dispenser and the patient [68]. It is for this reason that patients are highly concerned with finding a good private pharmacy service, rather than using a pharmacy service provided by public hospitals [67]. 

For many years, technology has been used in healthcare practices, including in laboratory investigations or as a method to create new diagnostic tools, such as stethoscopes [69]. As laboratory services are highly associated with patient satisfaction, the appropriate medical technology should be used as an aid for medical consultations. The use of technology facilitates a healthy consultation, but still patients are expecting in-depth laboratory investigations, which is consider as a central segment of the healthcare [70]. Regarding to the laboratories used in public hospital services in Pakistan, patients face problems, such as non-hygienic environments, late and fake results, uncompetitive staff, and a significant communication gap [7]. It is for these reasons that laboratory services directly impact on patient satisfaction. The prevalent issue is also highlighted and discussed in other part of the world such as Africa [71].

The present study fills the current gap in the literature by evaluating patient satisfaction in regard to pharmacy services, laboratory services, doctor–patient communication, and physical facilities. Doctor–patient communication and physical facilities did not have a significant impact on patient satisfaction in Pakistan. The physical facilities in OPD had a very small impact on satisfaction, which is proved from previous findings in India [72], South Korea [73], and Iran [74] where physical facilities had no significant relation with patient satisfaction.

For the sake of sustainability in the healthcare sector, governments should take serious steps to solve the issues in the healthcare sector, such as the creation of transparent policies, the provision of increased funding for physical infrastructure, and the involvement of all stakeholders in decision making. The healthcare administration should collaborate with the Provincial Health Department to digitize hospital records to decrease congestion and delays in laboratory services. Our findings will act as an important cue for healthcare administrators who could then be able to continuously maintain clean and attractive environments in hospital premises to retain the loyalty of patients. In order to maintain a better environment, vivid policy structures need to be created by the Government for uplifting the maintenance levels of their respective institutions. 

Our findings highlighted that hospital management teams need to focus on a timely delivery of services, proper communication, and the employment of staff that willingly care for patients. As the population increases, the level of patient dissatisfaction also increases. In order to cope this issue, the numbers of doctors and pharmacy staff working in hospitals should be increased. The physical environment plays an important role in patients’ perceived satisfaction. Therefore, hospital administrators should improve facilities, such as the availability of clean drinking water, and hygienic conditions in wash rooms and sitting areas. The results from this study are associated with quality features provided by WHO 2006 framework, which recommends that health services should be sustainable.

These findings are helpful for healthcare organizations to improve their service delivery. Healthcare organizations should adopt a redesigned service delivery procedure by giving it a patient-centric orientation. This strategy may result in better patient care and higher returns of trust for the organizations. We expect that this study will play a significant role in the literature of the healthcare sector. Scholars will get benefit from these findings and, we hope, will conduct similar research involving more parameters to improve Pakistan’s healthcare system.

## 6. Limitations and Future Research

There are some limitations to this study. One limitation is that this study was carried out in only the southern Punjab province of Pakistan. Another limitation is that this study used random sampling methods that involved a circumscribed number of patients. In future, research endeavors may be conducted by increasing the sample size and by using additional sampling methodologies to gain a deeper insight into the factors that affect patient satisfaction with the services provided in hospitals. Furthermore, attempts can be made by researchers to explore more critical factors, including a disease-wise investigation, which can add further understanding about the elements that influence patient satisfaction at the tertiary level of health problems.

## Figures and Tables

**Table 1:** The population of Punjab province and the three districts.

	Population	Area km^2^
Punjab Province	110,012,442	205,344
Bahawalpur District	3,668,106	24,830
Bahawalnagar District	2,981,919	8878
Rahim Yar Khan District	4,814,006	11,880

Source: Statistical Bureau of Punjab.

**Table 2:** Hospitals in the districts.

Sr. No.	Teaching Hospital	District Hospital (DHQ)	Tehsil Headquarter (THQs)	Rural Health Center (RHCs)	Basic Health Unit (BHUs)
1	1	0	4	10	72
2	0	1	4	10	101
3	1	0	3	19	104
	2	1	11	29	277

Source: Statistical Bureau of Punjab.

**Table 3:** Demographic characteristics.

Characteristics	Frequency	%
**Gender**		
Male	199	44.7
Female	246	55.3
**Age**		
Less than 20	36	8.1
20 to 29	53	11.9
30 to 39	93	20.9
40 to 49	137	30.8
50 and above	126	28.3
**Marital status**		
Married	243	54.6
Single	188	42.3
Divorced	12	2.7
Widow	2	0.4
**Occupation**		
Student	7	1.6
Government employee	45	10.2
House wife	117	26.2
Laborer	108	24.2
Agriculture	43	9.7
Un-employed	71	16.0
Retired	33	7.5
Other	21	4.6
**Education**		
No formal education	135	30.3
Primary/elementary school	145	32.6
Secondary/high school	106	23.8
College/university	24	5.3
Postgraduate	35	8.0
**Monthly income**		
Less than PKR 10,000	123	27.6
PKR 10,000 to 14,999	103	23.2
PKR 15,000 to 19,999	74	16.6
PKR 20,000 to 24,999	61	13.8
PKR 25,000 to 29,999	47	10.5
PKR 30,000 or more	37	8.3

**Table 4:** Descriptive statistics, Pearson’s correlations, and reliability coefficients among variables.

Variable	Mean	SD	1	2	3	4	5
Patient satisfaction	3.99	0.61	**(0.96)**				
Laboratory services	3.52	0.77	440 **	**(0.92)**			
Pharmacy services	4.15	0.66	0.445 **	0.339 **	**(0.93)**		
Doctor–patient communication	2.25	0.80	0.205 **	0.189 **	0.230 **	**(0.94)**	
Physical services	3.02	0.89	0.269 **	0.402 **	0.192 **	0.253 **	**(0.96)**

Notes 1: SD = Standard Deviation. Notes 2: Numbers in parenthesis are Cronbach’s Alphas (α). Notes 3: ** Correlation is significant at the 0.01 level (two-tailed).

**Table 5:** Composite reliability, convergent, and discriminant validity.

	CR	AVE	MSV	MAX (H)	PS	PHS	LS	PF	DPC
**PS**	0.965	0.753	0.230	0.976	**0.868**				
**PHS**	0.934	0.639	0.230	0.939	0.479 ***	**0.799**			
**LS**	0.928	0.685	0.202	0.935	0.450 ***	0.369 ***	**0.827**		
**PF**	0.964	0.844	0.148	0.984	0.267 ***	0.199 ***	0.385 ***	**0.919**	
**DPC**	0.942	0.804	0.063	0.974	0.220 ***	0.222 ***	0.192 ***	0.251 ***	0.897

Notes 1: PS: Patient Satisfaction, PHS: Pharmacy services, LS: Laboratory services, PF: Physical Facilities, DPC: Doctor–patient communication. Notes 2: CR = Composite Reliabilities, AVE = Average Variance Extracted, MSV = Maximum Shared Variance, MAX (H) = Maximal Reliability. Notes 3: Significance level: *** *p* < 0.001.

**Table 6:** Model fit statistics.

Absolute Model Fit Indices	
Chi Square	780.547
DF	415
Chi Square/DF	1.881
Standardized Root Mean Residual (SRMR)	0.042
Comparative Fit Index (CFI)	0.980
Normed Fit Index (NFI)	0.959
Tucker Lewis Index (TLI)	0.976
Relative Fit Index (RFI)	0.951
Incremental Fit Index (IFI)	0.980
Root Mean Square Error of Approximation (RMSEA)	0.045

**Table 7:** Results of the confirmatory factor analysis.

Construct/Factors	Items	Factor Loadings	Cronbach Alpha
Patient satisfaction			0.966
	Ps1	0.798	
	Ps2	0.817	
	Ps3	0.877	
	Ps4	0.893	
	Ps5	0.863	
	Ps6	0.723	
	Ps7	0.879	
	Ps8	0.876	
	Ps9	0.859	
Pharmacy services			0.931
	Ph1	0.780	
	Ph2	0.826	
	Ph3	0.796	
	Ph4	0.764	
	Ph5	0.797	
	Ph6	0.827	
	Ph7	0.795	
	Ph8	0.752	
Doctor–patient communication			0.942
	Dpc1	0.937	
	Dpc2	0.877	
	Dpc3	0.883	
	Dpc4	0.926	
Laboratory services			0.927
	Ls1	0.748	
	Ls2	0.851	
	Ls3	0.827	
	Ls4	0.845	
	Ls5	0.800	
	Ls6	0.770	
Physical services			0.968
	PF1	0.930	
	PF2	0.933	
	PF3	0.883	
	PF4	0.875	
	PF5	0.923	

**Table 8:** Multiple regression models (dependent variable: Patient satisfaction).

	β Coefficients		95.0% Confidence Interval for β			Collinearity Statistics	
	Β	T	Sig.	Lower Bound	Upper Bound	Tolerance	VIF
Constant		9.591	0.000	1.316	1.994		
1. Pharmacy services	0.287	6.328	0.000	0.158	0.300	0.766	1.306
2. Laboratory services	0.331	7.713	0.000	0.230	0.387	0.855	1.169
3. Doctor–patient communication	0.055	1.316	0.189	−0.021	0.105	0.900	1.111
4. Physical services	0.076	1.727	0.085	0-.007	0.113	0.806	1.241
Model summary *R* = 0.555, *R*^2^ = 0.308, *F* = 49.06, *p* = 0.000, Durbin-Watson (DW) = 2.01							
